# Catalytic promiscuity in the RNA World may have aided the evolution of prebiotic metabolism

**DOI:** 10.1371/journal.pcbi.1008634

**Published:** 2021-01-26

**Authors:** Dániel Vörös, Balázs Könnyű, Tamás Czárán

**Affiliations:** 1 Department of Plant Systematics, Ecology and Theoretical Biology, Institute of Biology, Eötvös Loránd University, Budapest, Hungary; 2 Evolutionary Systems Research Group, ELKH Centre for Ecological Research, Tihany, Hungary; 3 ELKH-ELTE Theoretical Biology and Evolutionary Research Group, Eötvös Loránd University, Budapest, Hungary; 4 Institute of Evolution, ELKH Centre for Ecological Research, Budapest, Hungary; Christian Albrechts Universitat zu Kiel, GERMANY

## Abstract

The Metabolically Coupled Replicator System (MCRS) model of early chemical evolution offers a plausible and efficient mechanism for the self-assembly and the maintenance of prebiotic RNA replicator communities, the likely predecessors of all life forms on Earth. The MCRS can keep different replicator species together due to their mandatory metabolic cooperation and limited mobility on mineral surfaces, catalysing reaction steps of a coherent reaction network that produces their own monomers from externally supplied compounds. The complexity of the MCRS chemical engine can be increased by assuming that each replicator species may catalyse more than a single reaction of metabolism, with different catalytic activities of the same RNA sequence being in a trade-off relation: one catalytic activity of a promiscuous ribozyme can increase only at the expense of the others on the same RNA strand. Using extensive spatially explicit computer simulations we have studied the possibility and the conditions of evolving ribozyme promiscuity in an initial community of single-activity replicators attached to a 2D surface, assuming an additional trade-off between replicability and catalytic activity. We conclude that our promiscuous replicators evolve under weak catalytic trade-off, relatively strong activity/replicability trade-off and low surface mobility of the replicators and the metabolites they produce, whereas catalytic specialists benefit from very strong catalytic trade-off, weak activity/replicability trade-off and high mobility. We argue that the combination of conditions for evolving promiscuity are more probable to occur for surface-bound RNA replicators, suggesting that catalytic promiscuity may have been a significant factor in the diversification of prebiotic metabolic reaction networks.

## Author summary

Complex biochemical machineries responsible for maintaining the correct ratio of enzymes and genes were highly unlikely to exist at the wake of life. Individual genes must have been subject to competition for resources of replication leading to the competitive exclusion between them, and thus to the loss of genetic information. A feasible scenario that avoids competitive exclusion requires the assumption of mandatory cooperation between the enzymes.

A potentially dynamically important but mostly neglected feature of RNA enzymes (ribozymes) is their capacity to catalyse more than a single reaction. Here, we analyse the possibility that this “promiscous” nature of prebiotic ribozymes could have helped the maintenance of early replicator communities cooperating in running a simple metabolism. To do so, we have implemented a spatially explicit computer model simulating the dynamics of replicating entities on a mineral surface–an extension of the Metabolically Coupled Replicator System including the possibility of multiple catalytic activities within the same replicator. Our results suggest that under realistic assumptions of replicator and metabolite mobility and feasible trade-off relations between different catalytic activities of the same RNA replicator molecule, catalytic promiscuity may have indeed helped booting up life through supporting the assembly of minimal metabolisms.

## Introduction

Based on our current knowledge about life it is safe to say that every recent living creature consists of one or more cells, each cell featuring three functional units/“subsystems” [1,2]: one storing the genetic know-how of self-maintenance and reproduction and passing it down the generations (genetic machinery), another one supporting the other two subsystems with material and energy (metabolism) [1], and a boundary subsystem (e.g. membrane) connecting the previous two to the external world while providing them with individual existence. It is unlikely that these three subsystems could have emerged and be functional in a coordinated manner from scratch in an abiotic environment all at once [3]. It seems much more probable that life had begun with a functional subset of the three subsystems–an *infrabiological system* [4]–and the rest was acquired by an evolutionary process later. Which subset may have been the one to kick-start abiogenesis is hotly debated. Almost any of the logically possible infrabiological systems has advocates among researchers of the origin of life, but the most widely accepted one is the genetics + metabolism first scenario: the RNA world hypothesis [5]. The RNA world owes its credibility to the fact that RNA molecules are capable of playing a dual chemical role: they can act as template-replicating information carrier molecules (genes) and as enzymes (*ribozymes*), with both functions embodied in a single chemical entity representing the genetic/metabolic infrabiological system. Now the question is whether RNA molecules can indeed run a genetic/metabolic machinery that is capable of self-maintenance and reproduction in an abiotic environment.

Apart from the many difficult problems related to the prebiotic supply of RNA building blocks [3,6–8] and template replication in the absence of specialized replicase enzymes [9–14], the RNA world hypothesis has to answer questions related to the stable maintenance of genetic information in RNA sequences that are sufficient to encode a working metabolism providing building blocks (nucleotides) for RNA replication, and a working replication machinery. Part of the problem is known as the Eigen paradox [15], which states that early (inefficient) enzymes (ribozymes) could not sustain long (RNA) sequences, but short sequences could not be efficient enzymes (e.g. replicases) [16–18]. The need for the maintenance and propagation of sufficient genetic information is pertinent to direct replication and metabolic contribution alike, and Eigen’s chicken-or-egg problem applies to both. The necessary amount of genetic information can be maintained by *i)* the coexistence of several short replicators each encoding a part of the whole genetic information, separately [15,19–22], by *ii)* gradually improving catalytic efficiency parallel with the length of single replicators [23], by *iii)* each replicator responsible for more than a single enzymatic activity, i.e., *catalytic promiscuity* [24–26] or by *iv)* a mixture of these [26,27].

The coexistence of different sequences (physically independent but functionally connected genes/ribozymes) is a feasible solution to Eigen`s paradox [15,19–21], but without a strict functional dependence between the replicators it leads inevitably to the problem of competitive exclusion: different species of RNA molecules cannot coexist on a single common resource (the common nucleotide pool) for long. A single member of the replicator community excludes all the others, and most of the information encoded in the community gets lost [28–30], in accordance with the Gause-principle [31,32]. Even worse, the winner of the competition will be the fastest replicating sequence, which is also the shortest in all probability. Eigen himself looked for a solution to the competitive exclusion problem by proposing the hypercycle model, but it has been proven to be evolutionarily unstable in all its implementations except when wrapped in growing and dividing membrane vesicles, which amounts to invoking the–unlikely–emergence of the gene/membrane infrabiological system from the very beginning without a functional coupling of the two subsystems and leaving metabolism out altogether [33].

**Fig 1:**
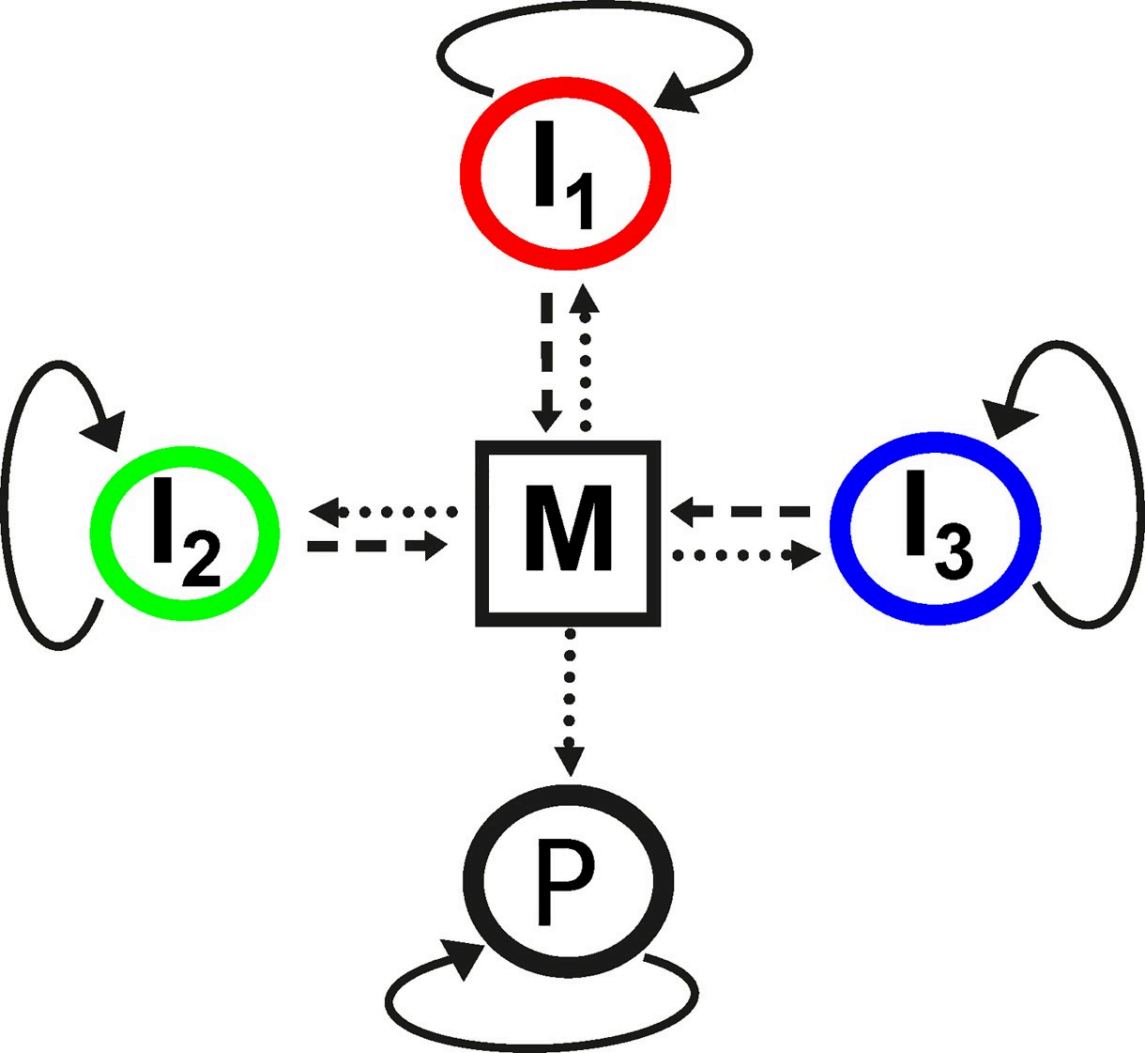
The Metabolically Coupled Replicator System concept. Metabolic replicators (*I_1_*, *I_2_* and *I_3_*) provide catalytic support (dashed arrows) to a common metabolism (*M*), which produces monomers (dotted arrows) for replicators to replicate (circular arrows). In this case, system size is *A* = 3 (number of catalytic activities needed for the metabolism). Parasites (*P*) use monomers supplied by metabolism in their replication, but they do not contribute to monomer production.

A physico-chemically more feasible class of replicator coexistence models is the Metabolically Coupled Replicator System (MCRS), which assumes the functional co-dependence of a number of different replicator species through the mandatory metabolic cooperation among all of them [20,34]. The replicators are assumed to be the ribozymes catalysing a minimal metabolic reaction network that produces monomers for their replication from external chemical resources (Fig 1). The complementary sequences of the ribozymes are their own genes [26,35,36]. The MCRS does not survive in a mean-field (well-mixed) setting, but it has been shown to be robustly coexistent both in the ecological and the evolutionary sense in spatial (e.g., surface-bound) models [20,33,34,37]: it resists competitive exclusion within the community, as well as the invasion of parasitic sequences produced by frequent mutations. The idea that surfaces may have had an important dynamical role in early replicator evolution [38] is supported by a number of empirical facts: RNA can be synthesized, attached to and survive on mineral surfaces [39,40], and the limited mobility of surface-bound molecules constrains their interactions to local neighbourhoods [3]. The dynamical reason behind the robust survival of the spatial MCRS is that the monomer supply of a replicator depends on the presence of all the other metabolically active ribozymes within its close neighbourhood. Fast replicating sequences are unable to outgrow and exclude slower members of the community because the fast species need the presence of the slow ones locally [20].

The MCRS scenario critically depends on the assumption of the availability of at least an inefficient template replication mechanism copying RNA sequences, the nature of which is still largely unknown. A feasible assumption would be that RNA replication was catalysed by other RNA molecules, and recent experimental work [9,14,41] points to the possibility of the imminent discovery of an RNA molecule capable of copying sequences at least as long as themselves with a sufficient accuracy, but we are not quite there yet. A structural problem of the MCRS is even more pressing, however: maintaining a working metabolism may need many more different catalytic activities than the surface-bound metabolic system can maintain against the assortment load (the number of copies lost by selection in metabolically incomplete neighbourhoods) [42]. The obvious solution to this problem would be chromosomatization (the containment of more than a single gene in one RNA sequence), but this implies yet another potentially complicated mechanism: the selective and restricted splicing of long RNA strands, which requires at least another ribozyme species, besides an accurate replicase capable of copying long chromosomes without too many errors. An alternative solution would be ribozyme “promiscuity”: catalytic activity of a sequence on more than a single substrate and/or reaction.

Recent protein enzymes are specific, highly evolved gadgets, each catalysing a single reaction on a specific set of substrates with high efficiency. But the features of prebiotic enzymes should not be derived from today`s extremely specialized enzymes. Prebiotic ribozymes may have been of very low efficiency and specificity, but they might have had more than a single activity [25,43]. It may occur in two different forms: *i)* substrate promiscuity (with the substrate-binding pocket of an enzyme accepting more different substrates) and *ii)* catalytic promiscuity (the ability of an enzyme to catalyse more than a single reaction type). We will use the term in its second meaning in this article. The dynamical importance of catalytic promiscuity lies in the opportunity to increase the number of catalysed metabolic reactions without increasing the number of replicator types involved [24]. This allows for evolving a more complex metabolic network without a prohibitively high assortment load on the MCRS. Besides, new single-activity catalysts are easier to evolve from promiscuous activities by specialization than it would be from a random sequence [25,44–46].

Theoretically, efficient catalytic activity implies complex 3D structure. The selective pressure for increasing the efficiency of a second active centre on the same sequence probably reduces the efficiency and the structural complexity of a resident catalytic site. This is a classical *trade-off* situation. Based on *in vitro* experimental evolution of contemporary enzymes the trade-off seems to be weak: increasing a secondary catalytic activity in a specialised enzyme induces only a small decrease in the primary activity [25,47].

The main goal of this project is to reveal the dynamical effects and the evolved patterns of catalytic promiscuity on the coexistence of metabolically active ribozyme replicators in the MCRS, assuming a few (3 or 5) potentially promiscuous catalytic activities in the basic surface-bound MCRS model by allowing multiple catalytic functions to emerge and evolve in any replicator molecule. Phenotypic mutations in concurrent catalytic functions and replicabilities are allowed within the limits of different trade-off constraints, and we ask under what environmental and trade-off conditions we may expect the evolution of generalist (promiscuous) and specialized ribozymes in the replicator community. Replicators losing all catalytic activities are parasites of the system; we also ask whether and when the replicator community resists destruction by its parasites. We show that promiscuity evolves in a wide range of the parameter space of the model, and the reason for this, along with that of the strong parasite resistance of the system, is straightforward.

## Methods

### The simulation model

**Fig 2:**
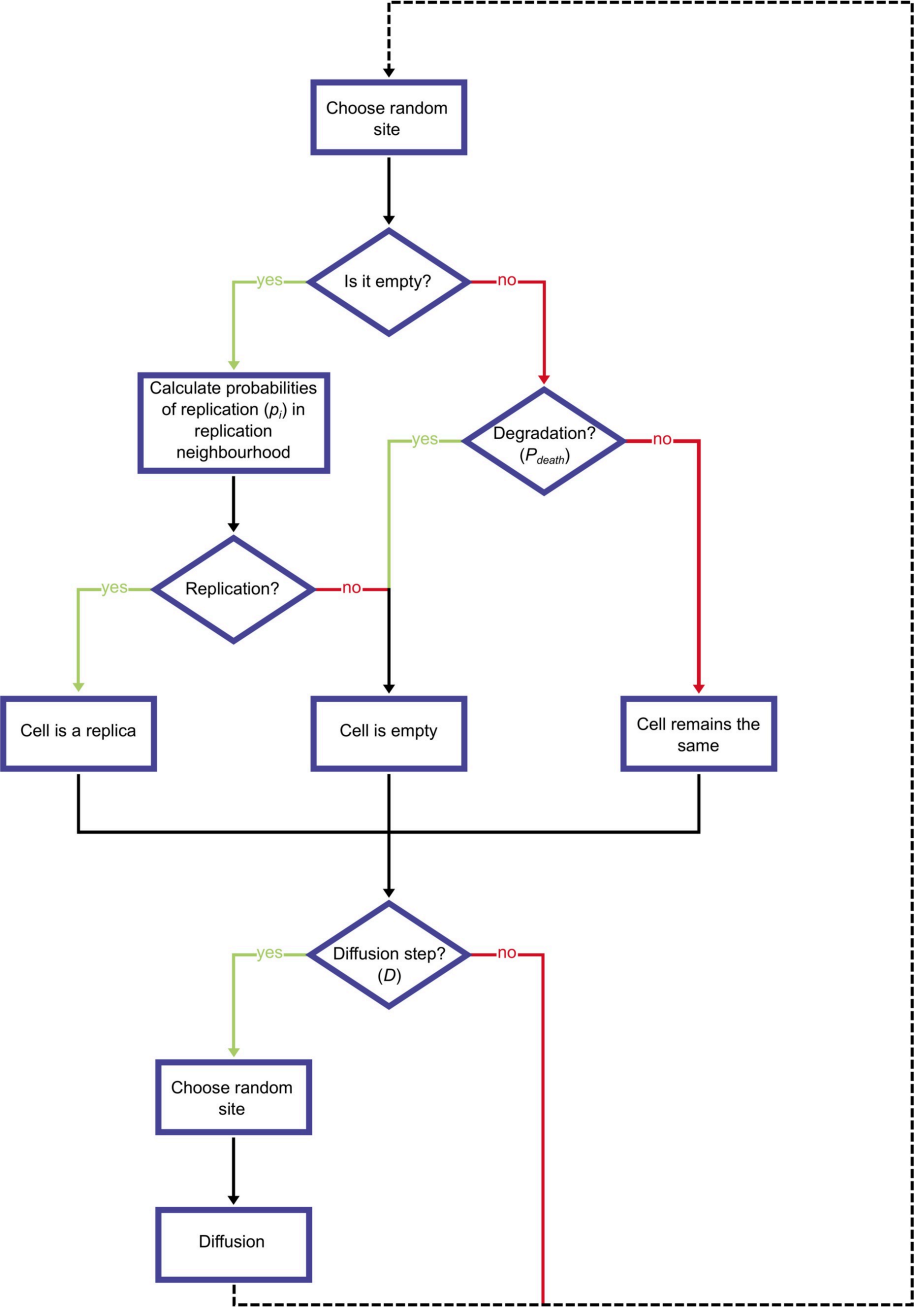
Simplified flow diagram of a single site update.

The model is a two-dimensional cellular automaton with 90 000 grid sites on a 300*x*300 square lattice representing prebiotic mineral surfaces to which replicators are attached. The grid is toroidal to avoid edge effects. Each grid site can be empty or occupied by a single replicator. Simulations lasted either until the 50 000^th^ generation or until the extinction of the last replicator, whichever occurred first. Simulations were initialised with 80% of the sites occupied by replicators of random properties (see later). During a generation each grid site was updated once on average, 90 000 updating steps taken in a random order on randomly chosen sites. Note that this random updating algorithm results in a Poisson distribution (of *λ* = 1) for the number of updates per site. An updating step may change the state of the focal site by one of the following events: *i)* no change; *ii)* if the site is occupied, the resident replicator degrades or *iii)* if the site is empty: one of the neighbouring replicators occupies it with a copy of itself (replication). The copy may be identical with the template, or it may be a mutant (see below). After each such demographic updating step a diffusion step may follow with probability *D*, resulting in the movement of replicators according to the Toffoli-Margolus algorithm (see below). Fig 2 is a flow diagram of a single updating step.

#### Degradation

RNA replicators may disappear from their habitat for several reasons in a prebiotic environment (e.g. hydrolysis, UV radiation, detachment from the surface), all of which result in the resident replicator disappearing from the grid for good with a constant rate *P*_*death*_ = 0.1.

#### Replication

**Fig 3:**
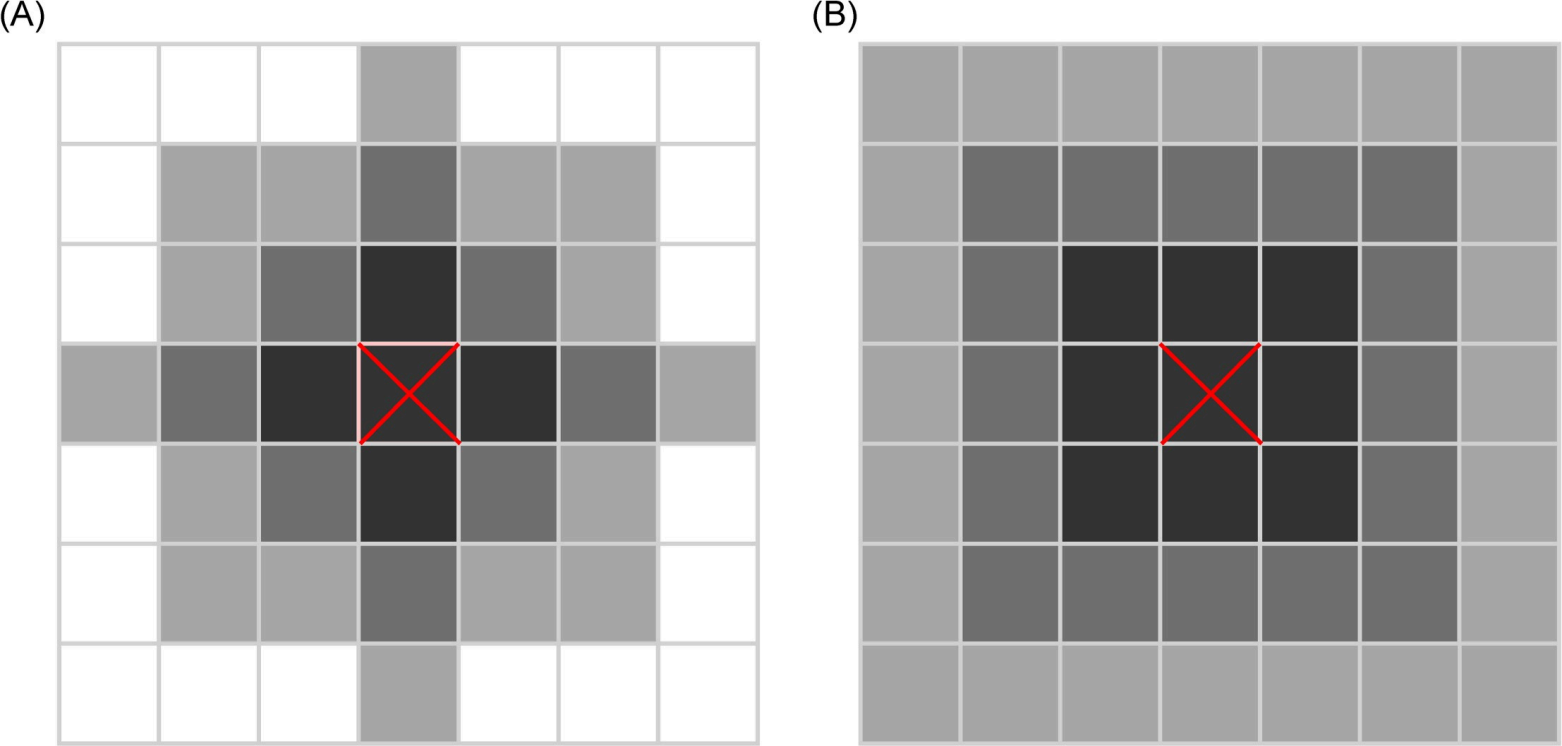
Neighbourhood types used in the simulations. **(A)** The von Neumann type neighbourhoods of the focal cell (red cross): black cells–the classical von Neumann neighbourhood with 5 cells; dark grey and black– 13 cells; light grey, dark grey and black– 29 cells. **(B)**. The Moore type neighbourhoods of the focal cell (red cross): black– 3*x*3 neighbourhood with 9 cells; dark grey and black– 5*x*5 neighbourhood with 25 cells; light grey, dark grey and black– 7*x*7 neighbourhood with 49 cells.

An empty grid cell might be occupied by a copy of an adjacent replicator from the replication neighbourhood of the empty site (see Fig 3). The success of replication depends on the individual “fitness” values (*W*_*i*_) of replicators *i* resident in the replication neighbourhood of the empty site:

$$W_{i}=k_{i}\times M_{i},$$ (1)

where *k*_*i*_ and *M*_*i*_ are the replicability and metabolic efficiency of the focal replicator *i*. Replicability *k*_*i*_ is the template quality of the replicator *i* to be copied, which mostly depends on the length and 3D structure of the template–short and loose sequences are easy to copy. Since the enzymatic activity of a sequence is higher for long and compact sequences, replicability and catalytic activity are in a trade-off relation. Replicability can take values between predefined minimum (*k*_*min*_ > 0) and maximum (*k*_*max*_) values (see details later).

The metabolic efficiency (*M*) of a replicator is the geometric mean of the sum of enzymatic activities (*E*_*a*,*l*_) within its metabolic neighbourhood (Fig 3):

$$M_{i}=\sqrt[{A}]{\prod_{a=1}^{A}\left(\sum_{l=1}^{N_{met}}E_{a,l}\right)}$$ (2)

*A* is the number of enzymatic activities necessary for the metabolic function (*A* = 3 or 5 in the simulations), and *N*_*met*_ is the number of cells within the metabolic neighbourhood of replicator *i*. The metabolic neighbourhood of a replicator is the concentric area around the replicator that has to contain all the necessary ribozyme activities in order to have sufficient monomer supply for its replication [20]. Any one of the enzymatic activities missing from the metabolic neighbourhood implies zero local metabolic help, and thus no replication.

The probability of replicator *i* to place a copy of itself onto a neighbouring empty site is

$$p_{i}=\frac{W_{i}}{C_{e}+\sum_{r=1}^{N_{repl}}W_{r}},$$ (3)

where *C*_*e*_ is the claim of an empty site to remain empty and *N*_*repl*_ is the size of replication neighbourhood around the empty site. The replication neighbourhood of a replicator defines the area within which its “offspring” molecules can bind. The probability of an empty cell to remain empty is

$$p_{e}=1-\sum_{i=1}^{N_{repl}}p_{i}.$$ (4)

### Catalytic activity transitions

Replicators are assumed to possibly acquire more than a single enzymatic activity, but within the same time step each ribozyme, even a promiscuous one, can catalyse only one reaction because of steric reasons [26,27,36]. Between time steps replicators can change their catalytic activity: they can be refolded into another structure with probability

$$P_{transition}=\frac{{\left(\prod_{i=1}^{B}E_{i}\right)}^{\frac{1}{B}}}{Max(E_{1},E_{2},\ldots ,E_{B})},$$ (5)

where *B* is the number of enzymatic activities of a certain replicator, and *E*_*i*_ is the value of activity *i*. *P*_*transition*_ is high if the activities are nearly equal (*E*_*1*_ ≈ *E*_*2*_ ≈… ≈ *E*_*B*_), and it is lower if activities are different (*E*_*k*_ >> *E*_*j*_; *j* ≠ *k*).

### Metabolic activity trade-off

**Fig 4:**
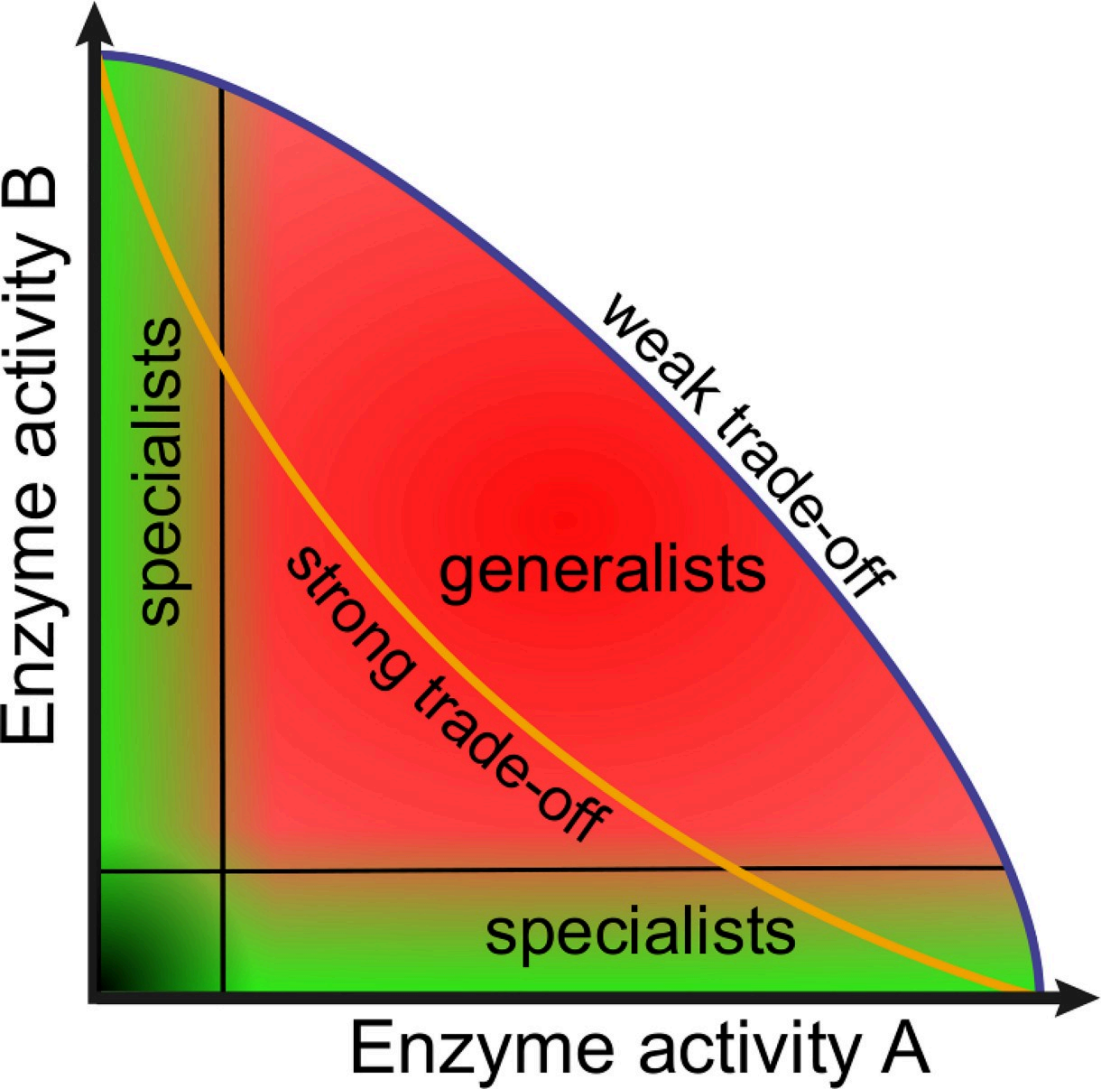
The types of replicators in the two dimensional space of enzymatic activities. Coloured regions represent parts of the phenotypic trait-space which replicators are permitted to occupy by the trade-off constraints. The concave orange line represents a strong trade-off, while the convex blue line stands for a weak trade-off between enzymatic activities A and B. Specialists (replicators in the green areas) have only one significant enzymatic activity; generalists (red area) feature both activities. Replicators of very weak or no catalytic activity are parasites (black area). While the generalist-specialists continuum is gradual, we distinguish them by artificial boundaries defined by a threshold value *m* = 0.01 (represented by black lines).

**Fig 5:**
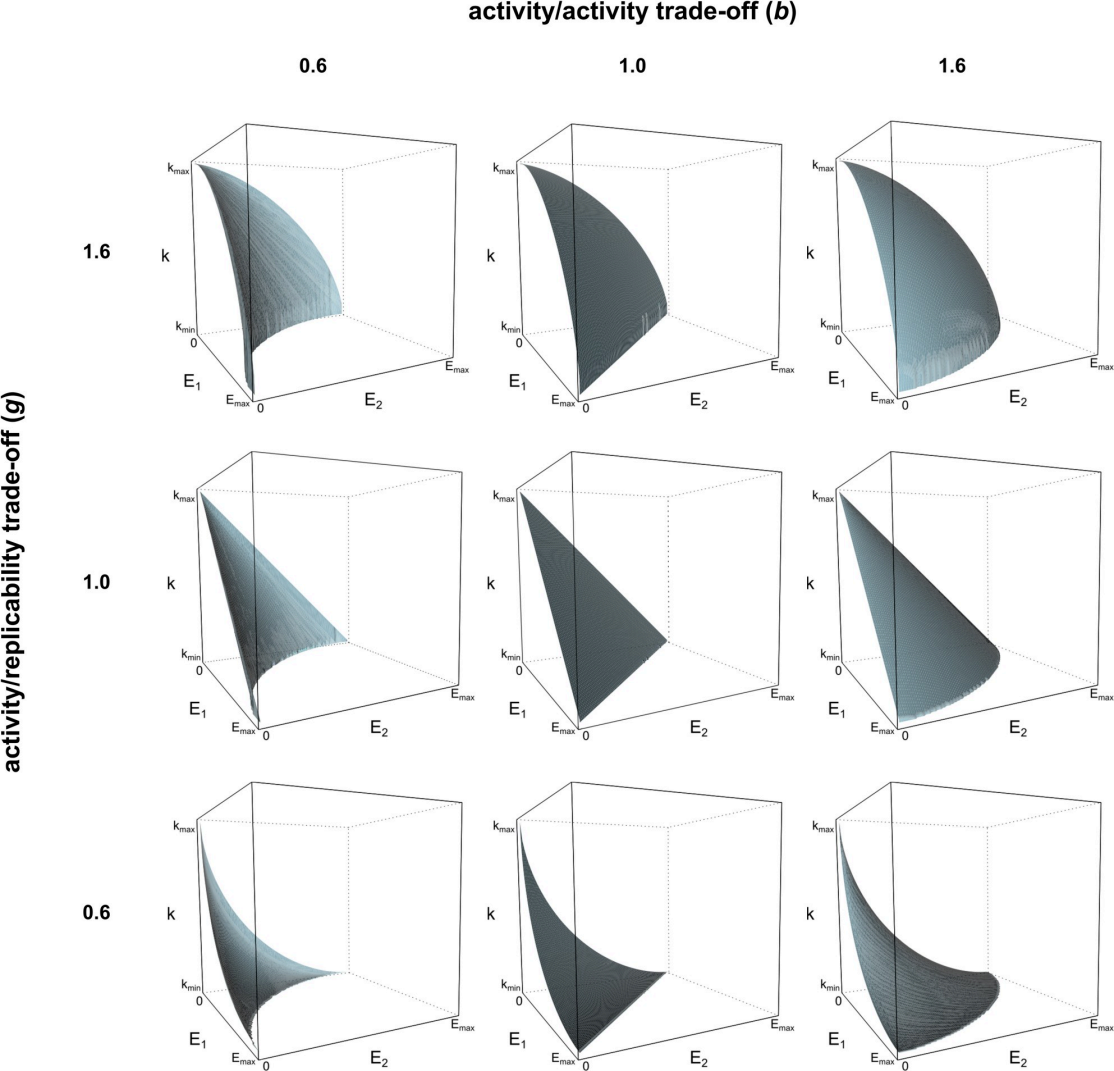
Trade-off surfaces with two enzyme activities. The surfaces delimit the section of the trait space that replicators can occupy in case of *A* = 2. The surfaces are calculated by Eqs 6 and 7; all replicators take trait values from below the trade-off surfaces shown. The axes are the two enzymatic activities (*E_1_* and *E_2_*) and replicability (*k*). Catalytic (activity/activity) trade-off differs in columns (left: strong, middle: neutral, right: weak) and activity/replicability trade-off differs in rows similarly (bottom: strong, middle: neutral, top: weak).

The enzymatic activities are in a trade-off relation with each other (see Fig 4): an enzymatic activity can increase only at the expense of the other activities accommodated by the same replicator, due to structural (folding) constraints:

$$\begin{array}{c} E_{max}^{b}\geq \sum_{a=1}^{A}E_{a}^{b},\\ E_{y}\leq {\left(E_{max}^{b}-E_{x}^{b}\right)}^{\frac{1}{b}}for\;any\;x,y\in A, \end{array}$$ (6)

where all the ribozyme activities (*E*_*x*_) are constrained by the others. *E*_*max*_ is the highest value that any of the enzymatic activities can reach, and *b* is the parameter defining the shape of the trade-off function (Figs 4 and 5) between the enzymatic activities. *b* is a continuous parameter, *b* = 1 representing a linear trade-off, *b* > 1 standing for weak and *b* < 1 for strong trade-off (Fig 4). In the model each of the enzymatic activity pairs has a trade-off relation with the same trade-off constant (*b*), assuming, for sake of simplicity, the same trade-off relation among the catalytic activities (Eq 6, second expression).

### Activity/Replicability trade-off

Enzymatic activities (*E*_*a*_) and replicability (*k*) are also traded off. The physico-chemical reason for this is that for an enzyme to be an efficient catalyst it has to be a relatively long sequence folded into a compact 3D structure. High replicability, on the other hand, requires the sequence to be relatively short and loosely folded, so that replicability and catalytic activity usually change in opposite directions in mutant copies, depending on changes in length and fold. The corresponding trade-off is defined in a way similar to that between the ribozyme activities (Eq 6), with a different trade-off constant (*g*). The trade-off surface for replicability *k* is scaled between a minimum (*k*_min_) and a maximum (*k*_max_) value, so that

$$k\leq {\left(E_{max}^{g}-{\hat{E}}^{g}\right)}^{\frac{1}{g}}\cdot \frac{k_{max}-k_{min}}{E_{max}}+k_{min},$$ (7)

where $\hat{E}={\left(\sum_{a=1}^{A}E_{a}^{b}\right)}^{1/b}$. The shapes of the trade-off surface are illustrated on Fig 5 for different *b* and *g* parameter combinations.

### Mutations

Phenotypic mutations occur with probability *P*_*mutation*_ = 0.01 during replication, changing the phenotypic traits of the copy: its enzymatic activities and its replicability may differ from those of the template. The replicability (*k*) and the enzymatic activities (*E*_*a*_) of a new replicator copy are mutated at random in such a way that the state of the mutant remains below the trade-off surface (see Eqs 6 and 7), i.e., neither trait of the mutant exceeds the corresponding coordinate of the bump point of the trait vector on the surface.

### Replicator classification

Based on enzymatic activities and replicability we classify different types of replicators (Fig 4):

*i) specialists*: replicators with exactly one enzymatic activity. The expressed ribozyme activity is high, the others are close or equal to zero, and replicability (*k*) is low;
*ii) generalists/promiscuous*: replicators with more than a single enzymatic activity. The number of significant activities is between two and the maximum (*A*). The expressed activities take intermediate values, and replicability is low to medium;
*iii) parasites*: replicators with very weak or no enzymatic activity, with *E_a_*≤0.1 for all

*a*∈(1,…*A*). Parasites’ replicability (*k*) may be high, close to *k*_*max*_ in many cases, but it may broadly vary under the trade-off surface; cf. Fig 5).

### Movement

The movement of the replicators is constrained onto the surface by adsorption which also limits their mobility. Surface diffusion is implemented using the Toffoli-Margolus algorithm [48], an elementary step of which consists of the rotation of a randomly chosen submatrix of 2*x*2 sites by 90° clockwise or anticlockwise with equal chance. The surface diffusion coefficient (*D*) is the number of such steps per demographic update step. This algorithm assumes equal mobility for all the replicators—this is a simplifying assumption not considering the dependence of adsorption on replicator length and structure [39,40]. *D* was either 0 (no replicator movement apart from copies placed on neighbouring empty sites) or 5 (intensive mixing).

## Results

The eco-evolutionary dynamics of the model are critically dependent on 6 of its 13 parameters (see Table 1): *i)* the trade-off among different enzymatic activities (*b*), *ii*) the trade-off between enzymatic activities and replicability (*g*) *iii)* replicator mobility (*D*), *iv)* the size of the metabolic neighbourhood (*N*_*met*_), *v)* system size (*A*) and *vi)* the attainable maximum of replicability (*k*_*max*_). The sensitivity of the system to the remaining 7 parameters has been shown to be marginal in previous work [34,42]; these parameters were kept constant throughout the present study. All simulations were run for 50 000 generations (4.5 billion update steps), which was sufficient to reach stationary states of replicator frequencies in all cases studied. Simulations with the same parameter settings were repeated at least 5 times, each with different random seeds, and their results were averaged. The replicators present on the surface at any time are classified into the categories set out in Fig 4, depending on their position within the phenotype space that is constrained by the actual trade-off parameters (*b* and *g*).

**Table 1:** Parameters of the model.

Parameter	description	values
*k*_*min*_	Minimum of replication rate	2.0
***k***_***max***_	**Maximum of replication rate**	**2.0; 2.5; 4.0**
***A***	**System size–the number of enzymatic activities required for metabolic function**	**3; 5**
*E*_*max*_	Maximum of enzymatic activity that a replicator can reach	10.0
***D***	**Replicator mobility–the number of movement steps per iteration**	**0; 5**
***N***_***met***_	**Metabolic neighbourhood size–the number of cells in the metabolic neighbourhood of a focal cell**	**5, 9, 13, 25, 29, 49**
*N*_*repl*_	Replication neighbourhood size–the number of cells in the replication neighbourhood of an empty cell	9
***b***	**Enzymatic trade-off exponent–the strength of trade-off between enzymatic activities**	**0.2 to 1.6 in steps of 0.1**
***g***	**Enzymatic activity–replication rate trade-off exponent–the strength of trade-off between enzymatic activity and replication**	**0.5, 1, 1.5**
*P*_*death*_	Probability of replicator decay/desorption	0.1
*P*_*mutation*_	Probability of mutation per replication	0.01
*C*_*empty*_	The claim of an empty site to remain empty	20.0
*n*_*grid*_	Lattice size	300*x*300

Parameters scanned during the simulations are in **boldface**.

The actual level of catalytic promiscuity *within the* replicator *community* present on the lattice at any time was measured by the average number of catalytic activities per replicator relative to the possible maximum (*A*) of the number of activities, which is called the *index of overall generalism* (*G*) and calculated as

$$G=\frac{\sum_{a=1}^{A}\left(a\cdot n_{a}\right)}{A\sum_{a=0}^{A}n_{a}},$$ (8)

where *n*_*a*_ is the number of replicators with *a* different activities within the community. Note that the *G* index is normalized into the [0, 1] range.

### Patterns of coexistence

**Fig 6:**
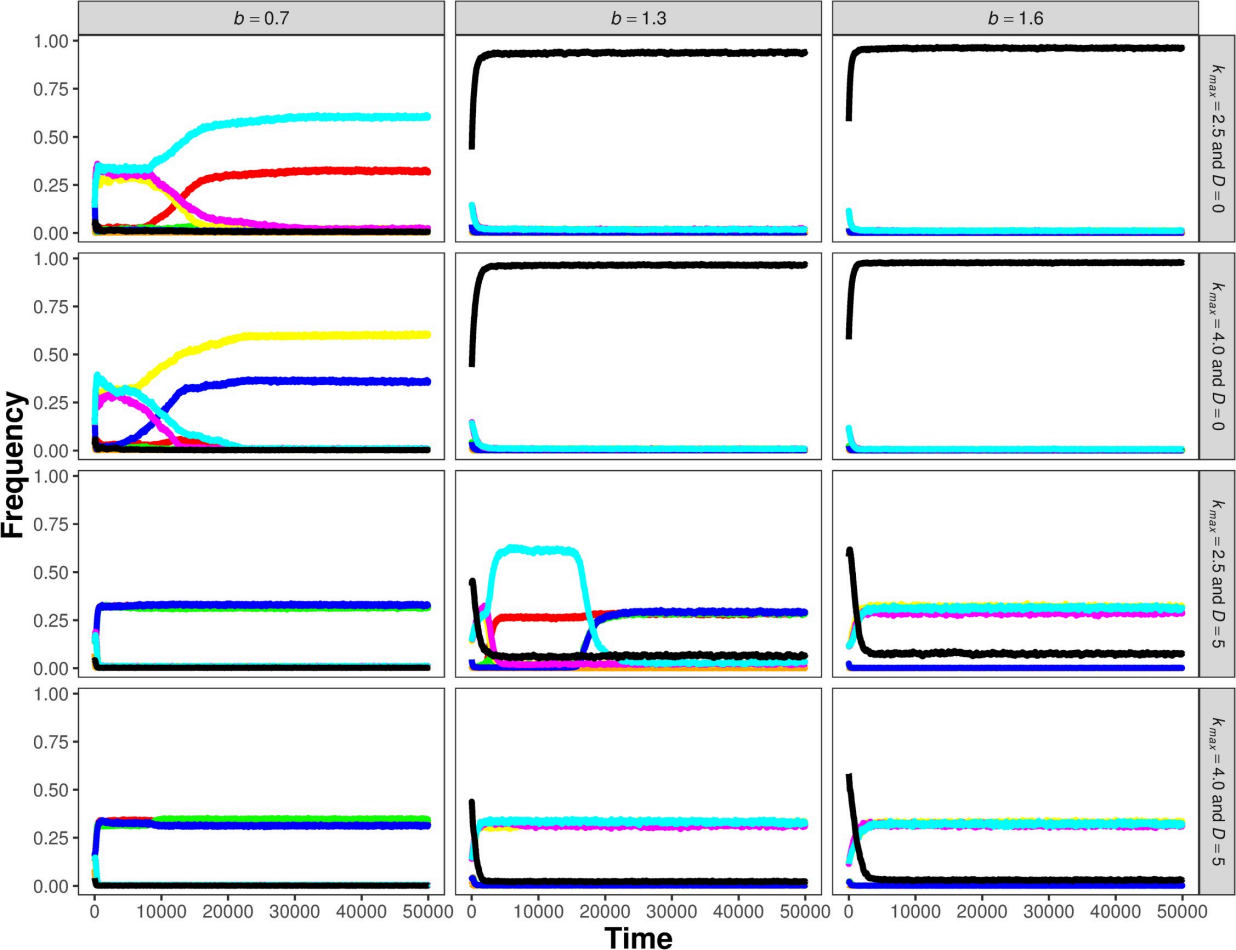
Time plots of the coexistence of different promiscuity patterns at different parameter settings. *b*, *k_max_* and *D* are specified on the top and right sides of the panels, other parameters: *A* = 3, *N_met_* = 5, *g* = 1.0. The colour code of replicators: orange - parasites, red–*E_1_*, green–*E_2_*, blue–*E_3_*, yellow–*E_1_/E_2_*, purple–*E_1_/E_3_*, turquoise–*E_2_/E_3_* and black–*E_1_/E_2_/E_3_*.

**Fig 7:**
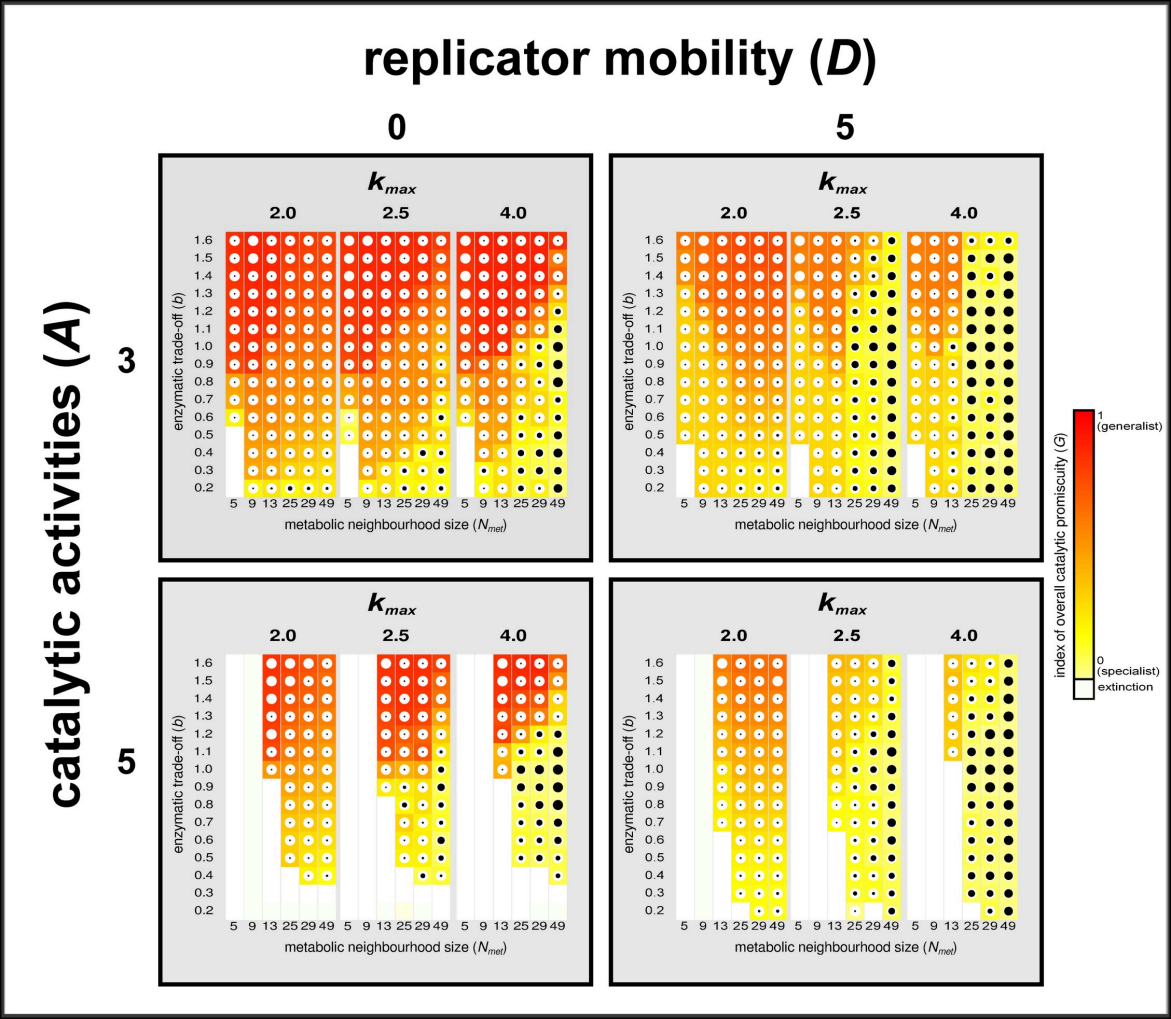
The effect of changes in key parameters of the system after 50 000 generations. This figure contains the results for simulations with *D* = 0 (left panels) and with *D* = 5 (right panels). The replication/catalytic activity trade-off parameter was *g* = 1 in every case. System size (number of enzymatic activity types in the system) is *A* = 3 (top) and *A* = 5 (bottom). Maximum replication rate (*k_max_*) and the size of the metabolic neighbourhood (see Fig 3) change from left to right. Square colour (within columns) represents the average of the relative (compared to system size) number of enzymatic activities per replicator after 50 000 generation (Eq 8), scaled from yellow (specialism) to red (generalism), represented by the index of overall catalytic promiscuity *G* (see Eq 8) within the whole replicator population. White squares indicate system extinction. White circles within coloured squares represent the whole replicator community, the black circles in them show the proportion of parasites (radii representing proportions). Each square / dot is calculated as the mean of at least 5 parallel simulations with the same parameter setting but different random number generator seeds.

**Fig 8:**
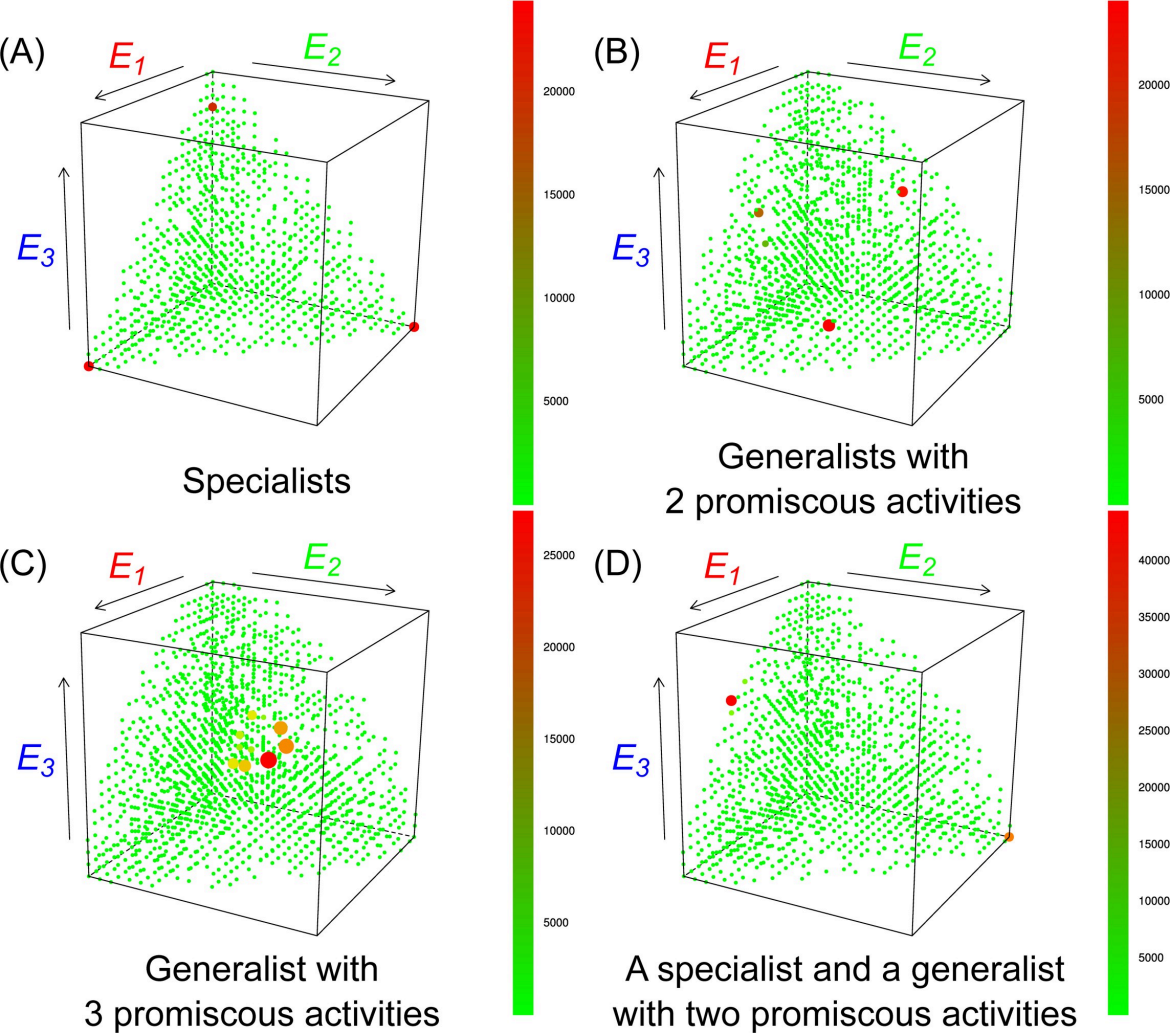
Promiscuity patterns of coexistence. The distribution of replicators under the trade-off surface of enzymatic activities after 50 000 generations at *A* = 3 and *g* = 1. Axes are scales of the three different enzymatic activities. Size and colour of dots represent the frequency of replicator groups with a certain enzymatic activity pattern. **(A)** a system dominated by specialists (*b* = 1.1, *D* = 5, *k_max_* = 2.0, *N_met_* = 5), **(B)** a system dominated by two-activity generalists (*b* = 1.5, *D* = 5, *k_max_* = 2.5, *N_met_* = 5), **(C)** a system dominated by three-activity generalists (*b* = 1.6, *D* = 0, *k_max_* = 2.0, *N_met_* = 5). On **(D)** a two-activity generalist type and its complementary specialist dominate the system (*b* = 1.4, *D* = 5, *k_max_* = 2.0, *N_met_* = 5).

Compulsory catalytic complementarity within metabolic neighbourhoods yields different coexistence patterns of replicators with different combinations of catalytic activity as illustrated by Figs 6–8. The evolved activity patterns depend on the six key parameters (system size *A*, catalytic trade-off *b*, catalytic/replicability trade-off *g*, replicator mobility *D*, metabolite diffusibility *N*_*met*_, and replicability range *k*_*max*_) of the model.

Simulations demonstrate that surface-bound replicators may coexist by adopting different promiscuity patterns for a wide range of the model parameters (Fig 6). This is not surprising: the MCRS model is known to maintain the robust coexistence of cooperating replicators in a substantial part of the feasible range of its parameter space [34] because of the constraint of mandatory local catalytic complementarity: all catalytically active replicators are assumed to play an essential role in monomer production through a complex reaction network which is implicit in the toy version of the model. Almost all chemical details (the reactants, the kinetics of the reactions and the topology of the network) of metabolism are unspecified except those (essentially *ecological*) features that are relevant with respect to the coexistence of the replicators: *i)* at least some of the metabolic reactions supplying monomers for replication are catalysed only by the replicators themselves (*A*, Fig 1), *ii)* the adsorption of the reactants and the products of metabolism (intermediates and monomers) to the surface is weak, therefore the range of their diffusion on the surface before desorption is limited, and *iii)* replicator mobility (*D*) is also restricted. The first two aspects depend on *the size of the metabolic neighbourhood* (*N*_*met*_).

### System size (*A*) and metabolic neighbourhood size (*N_met_*)

The metabolic neighbourhood of a replicator is the local area centered on its position on the surface within which all the mandatory catalytic activities have to be present so that the focal replicator be supplied by monomers for its own reproduction. Monomer production occurs if the metabolic neighbourhood is large enough to accommodate all the required catalytic activity types, which is obviously easier at *A* = 3 than at *A* = 5. This is why the range of the parameter space allowing for coexistence is narrower at five (*A* = 5) than at three (*A* = 3) compulsory catalytic activities (Fig 7, white squares extending to a larger part of the plots for *A* = 5). Since the number of catalytic activities fitting into a small metabolic neighbourhood is obviously larger if a single replicator can provide more than a single activity, a given metabolic neighbourhood size may, in principle, sustain a more complicated metabolic reaction network if catalytic promiscuity is allowed. The real limit on increasing the number of catalytic activities within the same macromolecule is the trade-off among them: due to constraints on folding the same sequence into different 3D structures, individual ribozyme activities decrease with each new catalytic function added.

Metabolic neighbourhood size (*N*_*met*_) is the area (number of sites) in which the intermediate metabolites and products (monomers) of the (implicit) reaction network have a high probability of remaining attached to the surface before dissociating and being lost for further metabolic reactions or replication. For simplicity it is assumed that metabolites and monomers crossing the border of the metabolic neighbourhood they belong to are lost (desorbed from the surface). We have shown earlier [20,34,42] that the coexistence of metabolically active replicators is an emergent feature of the MCRS, and it is based on the advantage of rarity: metabolic replicators with the lowest replication rate (*k*) have more chance to replicate because they have the highest chance of being complemented by the other, more common replicator species within their metabolic neighbourhood. Limited diffusion range for metabolites is a prerequisite for this advantage to occur: the model approaches its well-mixed mean-field version in the limit as *N*_*met*_ increases, and the mean-field approximation of the MCRS is known to collapse [20]. Then largest metabolic neighbourhood studied with the present simulations was 7*x*7 = 49 sites (cf. Fig 7)–above this metabolite diffusion range, the system breaks down.

### Replicator movement on the surface (*D*)

The anchoring of macromolecular replicators (as well as the temporary attachment of metabolites and monomers) to the mineral surface is the central assumption of the “prebiotic pizza” hypothesis [10,38]. Obviously, fixing all replicators to the surface with extremely limited movement (i.e., assuming *D* = 0) is detrimental for a system consisting of specialized, single-activity ribozymes, because it produces homogeneous, unviable patches of specialists lacking metabolic complementation (see the difference in the yellow area between *D* = 0 and *D* = 5 on Fig 7). At relatively high replicator mobility, however, complete metabolic neighbourhoods are recreated by spatial mixing, giving a chance for specialized ribozymes to survive. This positive effect of increasing *D* has been repeatedly demonstrated in earlier MCRS studies [20,34,42]. The flip side of intense mixing (high *D*) is the advantage it gives to parasites which in turn may destroy the system eventually, as explained in the next section. Allowing for catalytic promiscuity may alleviate the dynamical disadvantage of being tightly bound to the surface, which–with the replicators being charged RNA macromolecules–is straightforward to assume, given that clay minerals are also charged [39,49,50]. With more than a single catalytic activity per replicator, less intense mixing might have been sufficient to maintain functioning local metabolisms on such mineral surfaces.

### Parasites

Any cooperative system may suffer from parasites–so does the MCRS. The presence of parasites (replicators with no or marginal (<0.01) enzymatic activity and high replicability) in a metabolic neighbourhood may have a negative effect on the metabolic community: parasites may occupy many of the empty grid sites and become locally dominant within the “infected” neighbourhood. The system controls parasitic damage due to the negative density dependence of the parasitic subpopulation: local metabolic efficiency decreases with the number of parasites increasing, and finally local metabolism may collapse due to the exclusion of any one of the required catalytic activities which renders the metabolic neighbourhood incomplete, the parasite thus killing local metabolism and thereby committing suicide. However, at high metabolite mobility (with *N*_*met*_ high) parasites enjoy metabolic support even from more distant localities, and they can maintain large populations (see the black circles in yellow areas in Fig 7). Intensive replicator movement also helps parasites by destroying spatial segregation and distributing them into functional metabolic neighbourhoods.

### Evolutionary dynamics

Using many different combinations of the screened parameters we have recorded the evolutionary change in the evolvable features of the replicators populating the surface: the number and efficiency of their catalytic activities *E*_*a*_ (*a* = 1, …, *A*) as well as their replicabilities (*k*). These variables (*E*_*a*_ and *k*) represent the phenotypes of the replicators. They are subject to mutations within the limits set by trade-off constraints as explained in the Methods section and shown on Figs 4 and 5, at the fixed rate *P*_*mutation*_ = 0.01. Notice that with the focal features of the replicators mutable, the system’s dynamics will determine the distribution of these variables within the population, i.e., the patterns of catalytic activity and that of replicability within the replicator population is an *emergent feature* of the system. The time evolution of specialists and different generalists are shown on Fig 6, whereas the stationary replicator population structures seen after 50 000 generations of simulation with different parameter settings are summarized in Fig 7.

### Emergent patterns of catalytic activity

Fig 8 shows four characteristically different stationary metabolic/functional complementarity patterns at the end of the simulations (with the maximum number of enzymatic activities fixed at *A* = 3 for graphical illustration to be feasible).

A *specialist-dominated system* (Fig 8A) is one in which the dominant (most frequent) replicator types (red dots) provide a single, highly efficient enzymatic activity each. The frequencies of the three specialists are high and almost equal, the rest of the phenotypes (green dots) are their mutants. The emergence of specialist-dominated regimes is supported by high replicator mobility (*D*) and relatively large metabolic neighbourhoods (*N*_*met*_) through helping monomer production. Strong catalytic trade-off (small *b*) also favours specialization, especially at higher replicator mobilities, because two different specialists are more efficient catalysts than two promiscuous ribozymes each with the same two activities that are strongly traded off. Specialist-dominated systems are represented by yellow squares in Fig 7.

*Generalists with two activities* (Fig 8B) occur in all the three possible activity combinations, also at high and similar densities, surrounded by a cloud of mutants, typically at average (neutral) catalytic trade-off (*b*) and moderate replicator (*D*) and metabolite mobility (*N*_*met*_). Their catalytic activity patterns always complement each other within the population (see the time series of Fig 6). More generalist-dominated outcomes are represented by increasingly reddish squares in Fig 7.

The third, *classical generalist-dominated system* (Fig 8C) consists mainly of replicators providing all the three activities with nearly the same activity level and frequencies (reddish squares in Fig 7). For such complete promiscuity to emerge the catalytic trade-off must be weak (*b* > 1), and the mobility of both the replicators and the metabolites may be very small–the promiscuous system is not particularly sensitive to decreasing *D* and *N*_*met*_.

Finally, mixed *specialist-generalist systems* (Fig 8D) may evolve with different activity combinations upon the condition that local metabolic complementation remains complete. For example, an *E*_*2*_ specialist may be combined with an *E*_*1*_*/E*_*3*_ double generalist to provide a metabolically sufficient system at any location where they co-occur (see the time course of simulations on Fig 6). In this example (Fig 8D), the frequency of *E*_*2*_ specialists is lower than the frequency of generalists *E*_*1*_*/E*_*3*_, compensating the lower catalytic activity of generalist enzymes at the system level. Neutral trade-offs (*b* ≅ 1) and moderate replicator and metabolite mobility are the typical parameter settings at which this activity pattern may show up.

The different promiscuous outcomes cannot be sharply distinguished in Fig 7, the transition from one regime of activity pattern to another is gradual within the parameter space. As the catalytic trade-off becomes less restrictive (*b* increases), generalists tend to lose their handicap in catalytic activity but still enjoy the advantage of the easy assembly of metabolically complete local communities, which has a strong positive influence on system dynamics and the final pattern of catalytic activity mainly at low replicator mobility (*D* = 0 in Fig 7).

### System dynamics and stationary activity patterns

In general, most of the evolved communities become stable long before the 50 000^th^ generation, with the emerging dominant replicator species complementing each other metabolically. Occasional regime shifts do occur at some parameter combinations: one complete metabolic activity pattern may abruptly change to another, which then remains stable (see Fig 6), suggesting local and stochastic effects to play an important role in attaining one of the possible functional activity patterns on the community level. A simulation is considered to have reached its stationary state if no substantial change in the frequency distribution of replicator types occurs in a long time period.

*System size and relative catalytic promiscuity*: At larger system sizes (with *A* = 5) the functional complementary pattern of the system becomes more complicated and it may also change more dynamically in order to maintain the metabolic complementation at all times in any persistent system. Note, however, that although the number of actual catalytic activities per replicator, i.e., the absolute level of catalytic promiscuity, may in principle increase with *A*, the relative number of enzymatic activities per replicator in proportion to system size decreases at *A* = 5 compared to *A* = 3. (see Fig 7: red squares are paler for *A* = 5 than for *A* = 3). This may be due to the implicit constraint set by the catalytic trade-off parameter (*b*) on the system, even without considering the–probably rather strict–direct structural constraints of replicator length and folding.

### Controlling parasitism

Nothing, in principle, can prevent parasitic replicators (i.e., those with weak or no catalytic activity but high replicability) to occur as mutants, invade the surface, and destroy the metabolic cooperation of catalytically active replicators. It is their negative frequency dependence due to the damage they inflict upon local metabolism that controls their unconstrained takeover, and it does so in a very efficient manner as long as dynamical effects remain short distance. Parasitism can become fatal for the system only at large metabolite mobilities (*N*_*met*_) at which the negative frequency dependence effect becomes weak (and vanishes at the mean-field limit *N*_*met*_ = *Infinity*). The detrimental effect of increasing metabolite mobility through supporting parasites can be clearly seen on Fig 7 (black circles are bigger at high *N*_*met*_, also squares are getting less reddish as the number of activities per replicator decreases due to increasing parasitism).

The surprising trend of both strong and weak catalytic trade-offs (i.e., small and large *b*) being somewhat detrimental to parasites at larger metabolic neighbourhoods (Fig 7, smaller black circles at top and bottom of right columns) is connected to local metabolic efficiency. At strong trade-off the need for metabolic complementation using more copies of less promiscuous ribozymes leaves relatively little space for parasites, whereas at weak trade-off the low metabolic efficiency of more promiscuous ribozymes requires more copies of them to be present in order to maintain a sufficient local metabolism, which again leaves relatively few sites for parasites to occupy: local metabolisms with too many of them go extinct.

**Fig 9:**
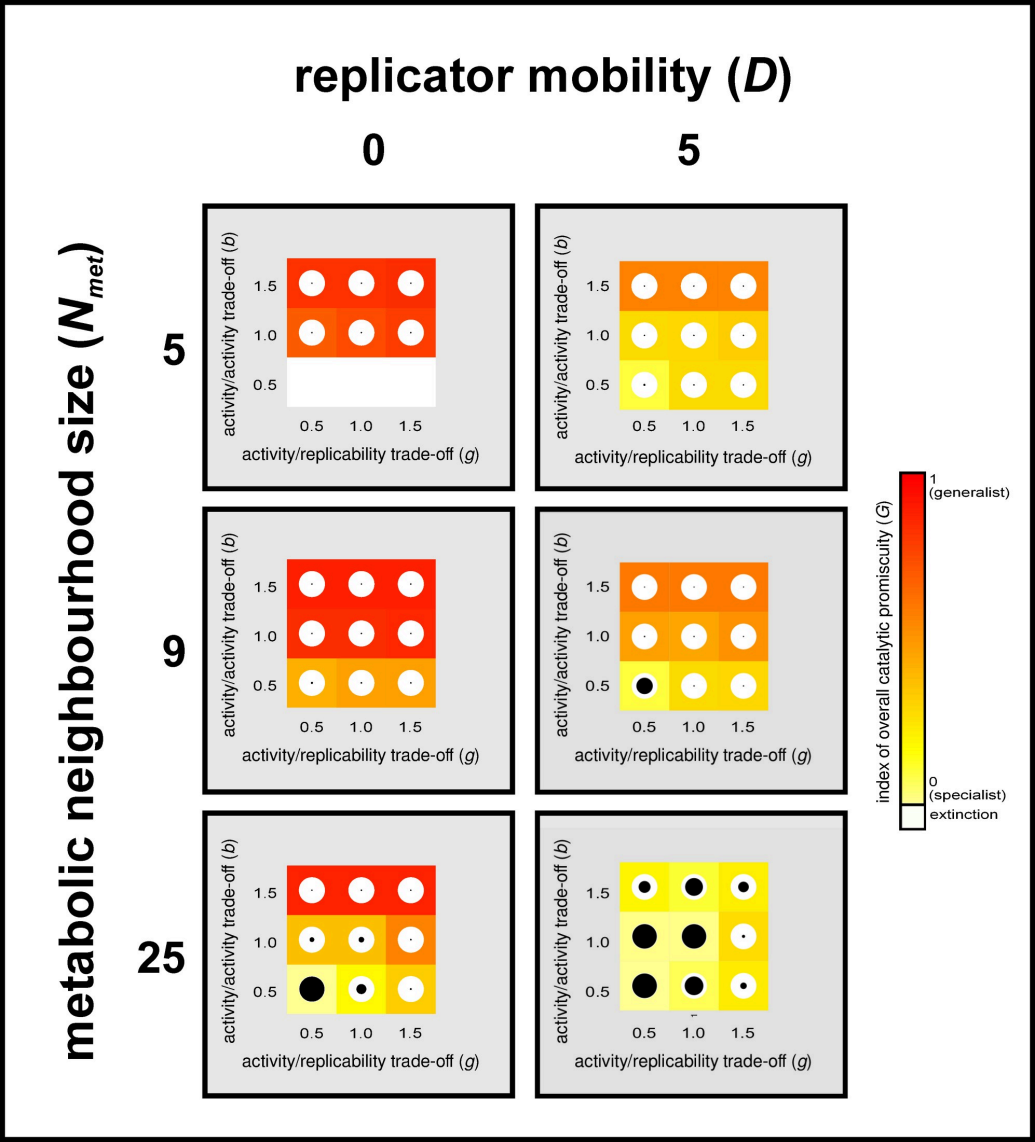
The effect of activity/replicability trade-off (*g*). This figure contains the results for simulations with *D* = 0 (left panels) and with *D* = 5 (right panels). The system size (number of enzymatic activity types in the system) is *A* = 3 and maximum replication rate is *k_max_* = 4.0 in all these simulations. Rows differ in metabolic neighbourhood sizes (top-down: *N_met_* = 5 (vonNeumann), *N_met_* = 9 (Moore), *N_met_* = 25 (5*x*5)). In the basic figures, the activity/replicability trade-off (*g*) gets weaker from left to right and the catalytic trade-off gets weaker from bottom to top. Square colour (within columns) represents the average relative (compared to system size) number of enzymatic activities per grid site after 50 000 generation (Eq 8), scaled from yellow (specialism) to red (generalism), that is calculated by the index of overall catalytic promiscuity *G* (see Eq 8) within the whole replicator population. White squares indicate system extinction. White circles within coloured squares represent the whole replicator community, the black circles in them show the proportions of parasites to the total number of replicators (radii representing proportions). Each square / dot is calculated as the mean of at least 5 parallel simulations with the same parameter setting but different random number generator seeds.

If the possible phenotypic variance of replicability is high (*k*_*max*_ >> *k*_*min*_) replicators tend to reduce their enzymatic activity in favour of their replication rate, to the extent allowed by the trade-off between replicability and catalytic activities (which is represented by *g* in Eq 7 and illustrated on Fig 5). High replicability is a direct fitness advantage, the most obvious feature on which efficient selection can operate [15,28]. It is prevented from increasing to its maximum only by the loss of the indirect fitness advantage that all replicators receive through compulsory local metabolic cooperation, which is constrained by the catalytic/replicability trade-off (*g*). Besides the obvious effect of helping parasitism, increasing *k*_*max*_ also helps specialization in cooperating replicators, but this latter effect is substantial only for high replicator mobility (c.f. Fig 7). Surprisingly, the shape of the activity/replicability trade-off function (*g*) plays no decisive role in the specialism/promiscuity/parasitism pattern (Fig 9), provided it is not prohibitively strict (which occurs at very low *g* values).

## Discussion

The MCRS model can provide two, somewhat different, plausible scenarios for the coexistence of enzymatically active replicators under prebiotic conditions: by storing information in a number of catalytically highly specific and efficient replicator types, or in a smaller number of less effective but promiscuous types. We have demonstrated that both these solutions are possible under the right assumptions regarding the surface mobility of the replicators and the metabolites they produce, and the trade-off relations between different replicator traits. Higher mobility (the movement of metabolites or enzymes) usually helps the assembly of specialist-dominated systems but also facilitates parasitism, while the possible gain in replication rate in exchange for a loss of enzymatic efficiency promotes promiscuous ribozymes and also parasitic sequences.

### Group selection

Since metabolic neighbourhoods are also the units of selection besides individual replicators, the MCRS represents a typical example for a group selection mechanism, the assortment load of which increases with system size *A*.

If all metabolic replicators accommodate the same number of enzymatic activities, then these enzymatic activities contribute to the common good at an equal measure. That is, they evolve the same catalytic efficiency and approach the same frequency, because the activities are weighted equally (see: Eq 3) and the price in replicability that replicators pay to express them is the same (*g* is equal to each activity) by definition. If replicators have different numbers of enzymatic activities in a stable metabolic neighbourhood, their replication rates and the strength of their enzymatic activities evolve to maintain a kind of dosage compensation of enzymatic activities through self-regulating their relative frequencies (see Figs 6 and 8D)–this is the basis of the very characteristic emergent stability feature of the model.

The key assumption of the RNA world hypothesis is the mandatory cooperation of different RNA replicators. This is an inevitable prerequisite to maintaining the coexistence and the evolvability of a replicator community with potentially different replicabilities, in order to avoid the competitive exclusion of all but the fastest replicating strain. An RNA community catalytically supporting its own monomer supply by the adoption of metabolic ribozyme activities meets this condition, provided that the ribozymes and the metabolites they produce are all immobilized to a certain extent as postulated in the surface-bound MCRS model, thus ensuring the efficacy of the local group selection mechanism without *ab ovo* assuming a highly specialized membrane system for its compartmentalization. In a mineral surface environment constraining replicator and metabolite mobility, enzyme promiscuity could have played an important role in the maintenance of a functioning metabolism, because it would have increased the number of metabolic reactions without increasing the inevitable assortment load of group selection on the system. With the feasible assumption that at the earliest phase of an RNA World there was no ribozyme capable of copying RNA strands the length of a multi-gene “chromosome”, natural selection for ribozyme promiscuity may have been the simplest way to increase the complexity of the metabolic system without extending the size of the RNA molecules embodying it.

### Trade-off relations

In this respect the critical assumption of the model is that the different catalytic activities of the same promiscuous replicator molecule are in a trade-off relation, mainly due to the–sometimes mutually exclusive–structural constraints that the different catalytic functions of a ribozyme molecule require. The question that needs closer empirical scrutiny is how constraining these constraints really are. An experimental study on ribozymes [25] suggests weak trade-offs between different catalytic activities of the same RNA sequence, which supports the idea that catalytic promiscuity may have played a role in maintaining prebiotic RNA communities, because weak catalytic trade-offs (larger *b* values in case of our model) help the spread of generalist replicators. At the same time, our conclusion that even relatively strong activity/replicability trade-offs (low *g* values in the model) can maintain functioning metabolic replicator communities–even if sometimes with a high load of parasites–suggests that the indirect advantage of metabolic complementation can, to a certain extent, offset the direct negative effects of parasitism on the coexistence of the community.

### The advantage of maintaining parasites

As explained earlier [20] the evolvability of any metabolic replicator system is critically dependent on the presence of a “parasitic” subpopulation that is kept at moderate frequencies by the system dynamics itself. Parasites are replicators with no dedicated function but potential subjects to mutational change to any possible new metabolic (or, in fact, any other beneficial) function that may increase the group fitness of the replicator community [34]. In this manner, the activity/replicability trade-off also works for the evolutionary improvement of surface-bound communities of metabolic replicators, by maintaining the pre-adapted “quasispecies” of a parasitic replicator population that is free to parse the sequence space for functions useful for the whole replicator community.

Finally, note that most laboratory experiments on catalytic promiscuity have been carried out on protein enzymes so far, for which switching to a different secondary structure is no option. Therefore, what is usually found experimentally for protein enzymes is either substrate promiscuity (an enzyme catalyses the same chemical reaction on different substrates) or catalytic homology (different enzymes acting on the same substrate). The type of catalytic promiscuity we have studied is different in that we assume ribozymes capable of re-folding into different secondary structures, resulting in different catalytic entities potentially acting on very different substrates. To what extent this assumption is feasible is to be determined by future experiments on catalytically active RNA focusing on their potential for catalytic promiscuity.
